# ^11^C-Methionine-PET: A novel and sensitive tool for monitoring of early response to treatment in multiple myeloma

**DOI:** 10.18632/oncotarget.3053

**Published:** 2015-01-20

**Authors:** Katharina Lückerath, Constantin Lapa, Christa Albert, Ken Herrmann, Gerhard Jörg, Samuel Samnick, Herrmann Einsele, Stefan Knop, Andreas K. Buck

**Affiliations:** ^1^ University Hospital Wuerzburg, Department of Nuclear Medicine, Wuerzburg, Germany; ^2^ University Hospital Wuerzburg, Department of Internal Medicine II, Division of Hematology and Oncology, Wuerzburg, Germany

**Keywords:** Multiple Myeloma, ^11^C-Methionine-PET, treatment response, molecular imaging

## Abstract

Multiple myeloma (MM) remains an essentially incurable hematologic malignancy. However, new treatment modalities and novel drugs have been introduced and thus additional tools for therapy monitoring are increasingly needed. Therefore, we evaluated the radiotracers ^11^C-Methionine (paraprotein-biosynthesis) and ^18^F-FDG (glucose-utilization) for monitoring response to anti-myeloma-therapy and outcome prediction. Influence of proteasome-inhibition on radiotracer-uptake of different MM cell-lines and patient-derived CD138^+^ plasma cells was analyzed and related to tumor-biology. Mice xenotransplanted with MM.1S tumors underwent MET- and FDG-μPET. Tumor-to-background ratios before and after 24 h, 8 and 15 days treatment with bortezomib were correlated to survival. Treatment reduced both MET and FDG uptake; changes in tracer-retention correlated with a switch from high to low CD138-expression. In xenotransplanted mice, MET-uptake significantly decreased by 30–79% as early as 24 h after bortezomib injection. No significant differences were detected thus early with FDG. This finding was confirmed in patient-derived MM cells. Importantly, early reduction of MET- but not FDG-uptake correlated with improved survival and reduced tumor burden in mice. Our results suggest that MET is superior to FDG in very early assessment of response to anti-myeloma-therapy. Early changes in MET-uptake have predictive potential regarding response and survival. MET-PET holds promise to individualize therapies in MM in future.

## INTRODUCTION

Multiple myeloma (MM) is a hematological malignancy arising from clonal expansion of post germinal center B cells (plasma cells). These B cells are characterized by markedly increased production of aberrant immunoglobulins (Ig; M-protein), which are secreted but also accumulate intracellularly. Although recent years have brought great progress in terms of treatment options including reversible and non-reversible proteasome inhibitors (e.g. bortezomib, carfilzomib and ixazomib), immunomodulatory drugs (e.g. thalidomide, lenalidomide and pomalidomide) or stem cell transplantation, almost all MM patients relapse (1, 2). Thus, MM remains an essentially non-curable disease, at least partly due to striking inter- and intra-patient heterogeneity involving multiple MM clones and rapid evolution of resistance (3, 4).

The role of imaging in MM remains to be clarified. Current diagnostic and staging strategies rely on structural imaging techniques such as whole body X-ray or computed tomography (CT). Both modalities aim at the detection of anatomical lesions, but fail to give information on disease activity. Thus, they are not suitable to monitor treatment response. Several studies have shown the usefulness of molecular imaging techniques such as positron emission tomography (PET) or PET/CT using the radiolabeled glucose analog 2-deoxy-2-[^18^F]fluoro-D-glucose (^18^F-FDG) for diagnosis, staging and prognostication of MM, leading to its implementation into the Durie/Salmon PLUS staging system (5–11). In addition, PET/CT was recently reported to be a very useful tool for monitoring of anti-myeloma therapy (12). However, reduced sensitivity and specificity of FDG (e.g. diffuse bone marrow infiltrations [false negative] or inflammatory lesions [false positive]) has been reported (13). In order to improve and individualize patient management, there is clearly a need to develop and validate novel tracers. Requirements for an ‘ideal’ tracer would include its ability: to differentiate between active and non-active MM manifestations (both intra- and extramedullary lesions); importantly, to provide a link to the underlying biology and/or metabolism; to depict tumor heterogeneity and/or to identify MM subtypes; to allow for an early diagnosis and to monitor response to therapy as early as possible.

We previously demonstrated a significantly higher retention of the radiolabeled amino acid *L*-methyl-[^11^C]-methionine (^11^C-MET) and an association of MET-uptake with Ig light chain levels in biologically diverse myeloma cell lines and patient-derived CD138^+^-plasma cells (14). This study aimed at the *in vivo* validation of MET as radiotracer and biomarker for MM. In addition, the versatility of MET and FDG for monitoring treatment response to different proteasome inhibitors was assessed in human MM cell lines, primary patient-derived CD138^+^-plasma cells and xenografted mice. Lastly, the predictive potential of these radiotracers regarding response and survival was assessed.

## RESULTS

### Monitoring response to proteasome inhibitors in MM cell lines using MET or FDG

**Figure 1:**
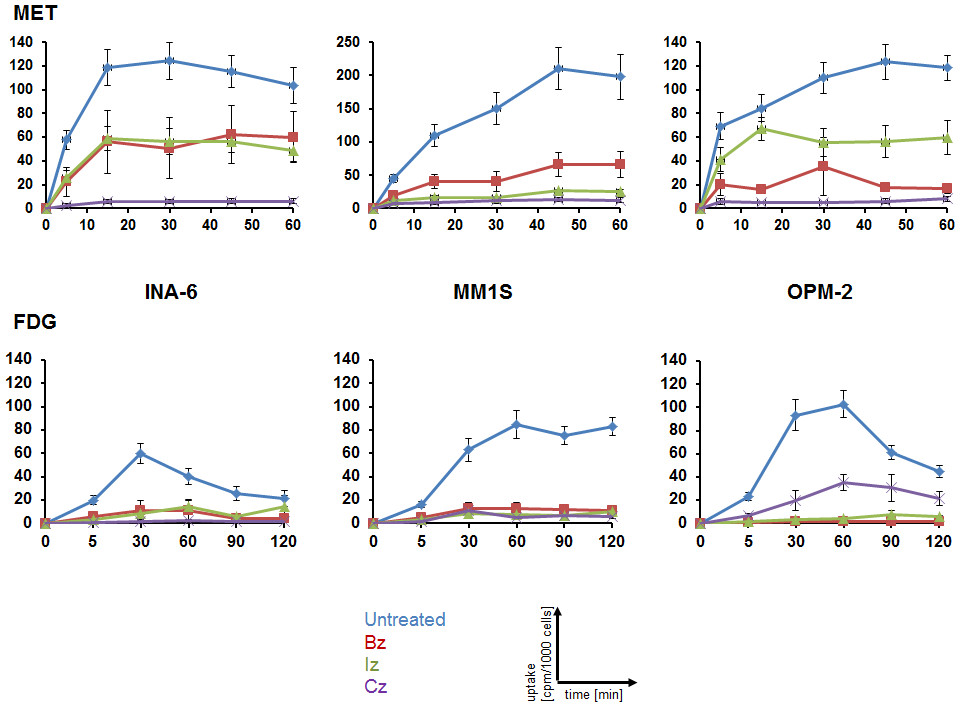
Monitoring response to proteasome inhibitors in MM cell lines using MET or FDG Cells were treated for 48 h with either proteasome inhibitor or left untreated before intracellular radioactivity after incubation with MET (top) or FDG (bottom) was quantified using a gamma-counter. Relative uptake of background- and decay-corrected triplicate-samples was expressed as cpm per 1000 cells (mean ± sem; *n* = 4).

To evaluate the suitability of MET and FDG for monitoring response to anti-MM therapy, MM cell lines were treated with either of three proteasome inhibitors, bortezomib (Bz; Velcade^®^), ixazomib (Iz; MLN9708) or carfilzomib (Cz; Kyprolis^®^). 48 h after drug addition, the capacity to store MET or FDG, respectively, was assessed in uptake experiments. In agreement with our previously published data (14), MET uptake in untreated control cells was higher than that of FDG. Comparing untreated with treated cells, a significant reduction in tracer-retention could be observed for all treatments at any time points analyzed with remaining MET-uptake slightly higher than that of FDG (Figure 1).

### Changes in tracer uptake upon proteasome inhibition are associated with CD138 cell surface levels

**Figure 2:**
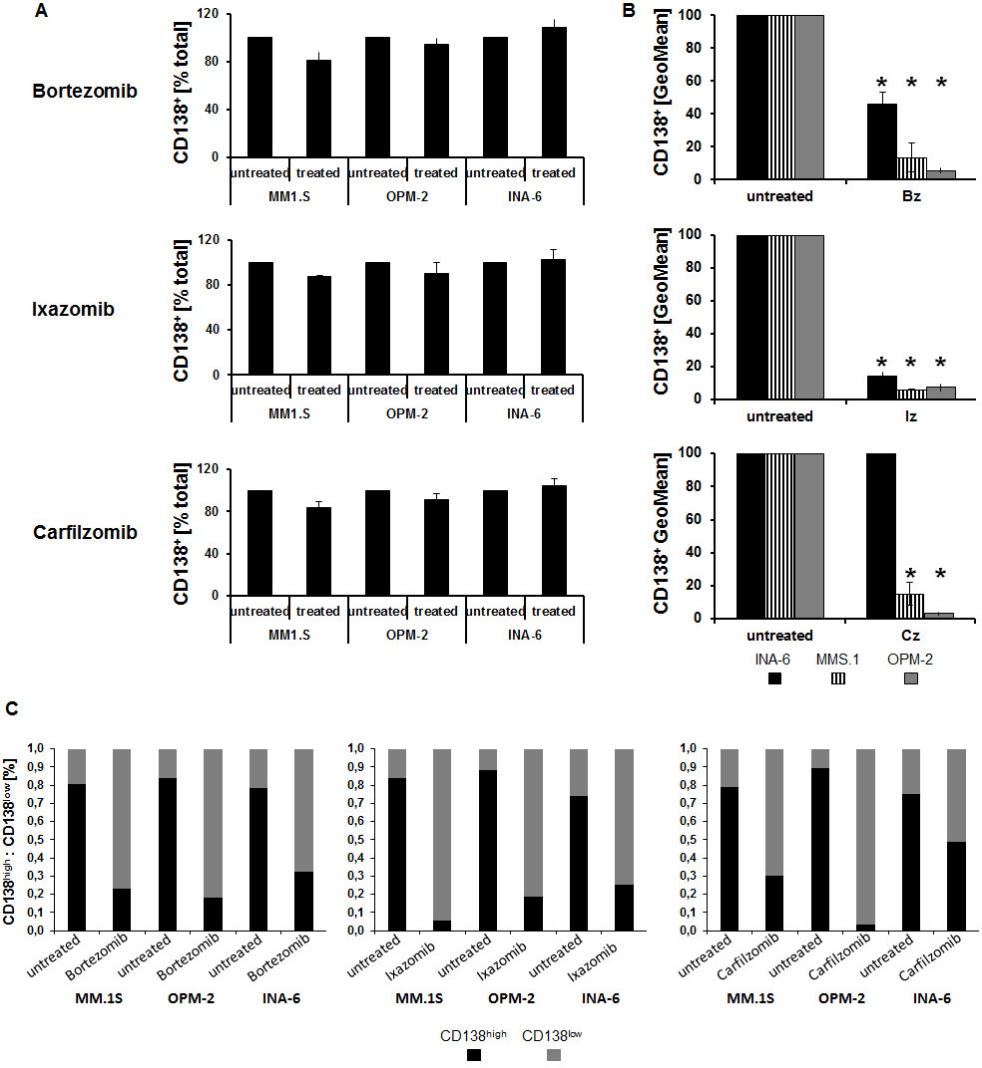
Changes in tracer uptake upon proteasome inhibition are associated with CD138 cell surface levels Flow cytometric quantification of the percent of CD138^+^ cells (percent of total; **(A)**), extent of CD138 expression (geometric mean fluorescent intensity, GeoMean; **(B)**) and percent CD138^high^ and CD138^low^ MM cells, respectively, **(C)** is shown as background-corrected means ± standard deviation, related to that of untreated controls (*n* = 5). Asterisk indicate statistically significant differences (*p* < 0.02).

Analysis of the MM marker and adhesion molecule CD138 at the cell surface demonstrated an almost identical fraction of CD138 expressing cells following incubation with either proteasome inhibitor (Bz, Cz, Iz) when compared to untreated controls (Figure 2A). However, the extent of CD138 expression was significantly reduced (*p* < 0.02) (Figure 2B); a more detailed evaluation revealed that the ratio of CD138^high^ to CD138^low^ MM cells reversed (Figure 2C). This reversal was positively associated with radiotracer uptake: the larger the reduction in tracer uptake, the less CD138 was present at the cell surface of MM cell lines (Supplementary Figure 1A; see the Supplemental Data Set link at the top of the online article). Results from ELISA-assays suggested that the loss could only in part be explained by enhanced shedding of CD138: while the amount of CD138 in the medium of OPM-2 cells treated with Bz increased 2-fold (compared to untreated cells), it was about the same in MM.1S and half as much in INA-6 (Supplementary Figure 2).

### Treatment-induced alterations of further hallmarks of MM biology do not parallel changes in tracer uptake

MM diagnosis and follow-up include analysis of various markers, including levels of Ig light chains in the serum of patients and assessing the proliferation rate in bone marrow biopsies. Based on that and our previously reported potential association of intracellular Ig light chains with basal MET uptake (14), treatment-induced changes in intracellular Ig light chains as well as in proliferation were determined.

**Figure 3:**
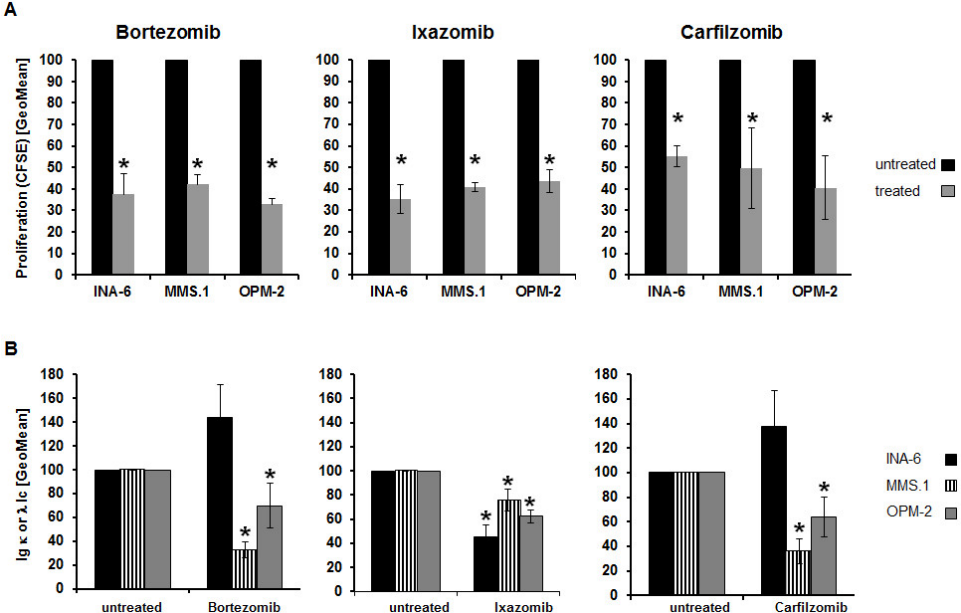
Treatment-induced alterations of further hallmarks of MM biology do not parallel changes in tracer-uptake **(A)** Relative proliferation rate as assessed by CFSE dilution (GeoMean; *n* = 5). **(B)** Intracellular levels of either λ- (MM.1S, OPM-2) or κ- (INA-6) immunoglobulin light chains were determined by FACS analysis (GeoMean) using anti-Ig λ-FITC- and anti-Ig κ-APC antibodies (*n* = 5). Background-corrected means ± standard deviation related to that of untreated controls are shown. Asterisk indicate statistically significant differences (*p* < 0.02).

All three proteasome inhibitors uniformly reduced the proliferative activity of MM cell lines by 50–60% compared to untreated controls (*p* < 0.02) (Figure 3A). However, the extent of this reduction did not parallel the change in uptake of either MET or FDG (Supplementary Figure 1B).

Likewise, levels of intracellular Ig light chains were significantly (*p* < 0.02) lower in MM.1S and OPM-2 cells following drug exposure, but the extent of reduction of Ig levels and tracer uptake, respectively, did not correlate. In INA-6 cells Ig light chain levels only decreased after exposure to Iz (~50%), while Bz and Cz resulted in approximately 1.4-fold higher levels (Figure 3B, Supplementary Figure 1C).

### Early assessment of response to Bz in CD138^+^-plasma cells using MET or FDG

**Figure 4:**
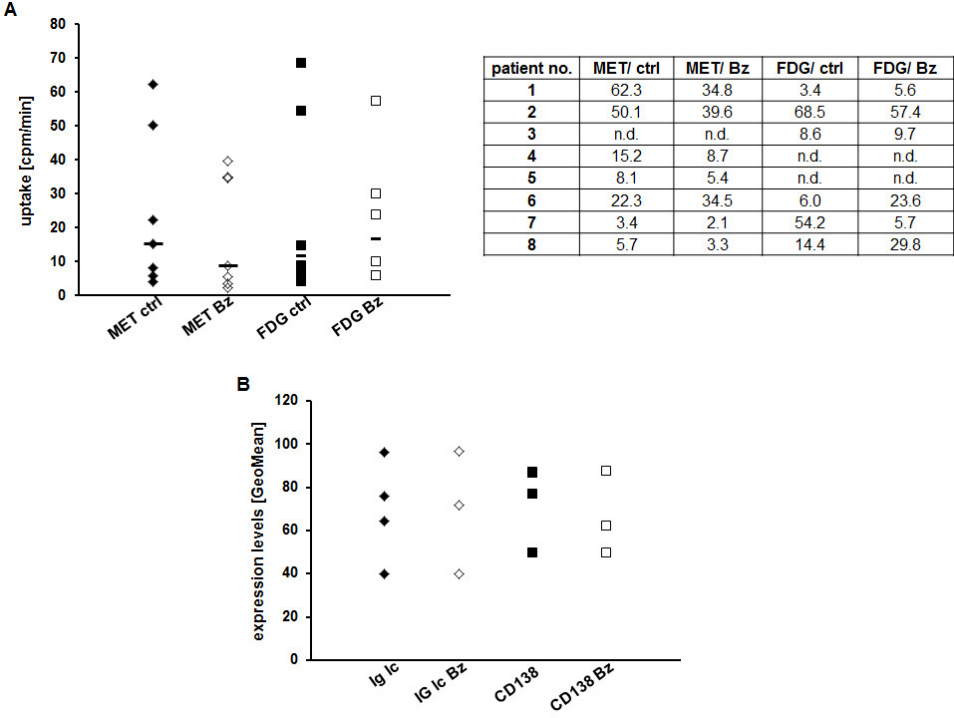
Assessing early response to Bz in CD138+-plasma cells using MET or FDG **(A)** CD138^+^-plasma cells from individual patients were or were not treated with Bz for 24 h, subsequently incubated with either FDG or MET for 60 min and intracellular radioactivity was quantified using a gamma-counter. Relative uptake of background- and decay-corrected samples was expressed as mean cpm per 1000 cells. Horizontal lines indicate the median in each group. The table gives the uptake values (cpm/1000 cells) for each patient. **(B)** Cell surface expression of CD138 and intracellular levels of Ig light chains in control and treated primary MM cells are shown as GeoMean (*n* = 4).

To validate our findings in a more physiological setting, we isolated primary CD138^+^-plasma cells from MM patients (see Supplementary Table 1 for patients' characteristics), split the samples into four groups and analyzed radiotracer uptake: (i) untreated/MET, (ii) untreated/FDG, (iii) 24 h Bz/FDG, (iv) 24 h Bz/MET. In agreement with the results obtained in MM cell lines, addition of Bz decreased MET retention markedly in 6/7 cases (remaining sample: higher uptake in presence of Bz); in contrast, FDG uptake increased in 4/6 samples and decreased in only 2/6 (Figure 4A). In the 4 samples that contained enough cells for further FACS analyses, neither levels of intracellular immunoglobulin light chains nor expression of CD138 were impacted at this early time point (24 h drug exposure) (Figure 4B).

### MET-PET reveals very early treatment response *in vivo*

As radiotracers underlie an *in vivo* metabolism which might impact their uptake by tumors, we investigated MET as well as FDG retention in Nod.Scid mice bearing MM.1S tumor xenografts before and 24 h after treatment with Bz. Prior to treatment, PET-imaging with MET, in clear contrast to FDG, resulted in high tumor-to-background ratios (median of mean TBR: MET 3.98, range 1.1–8.0; FDG 1.92, range 0.7–5.8; *n* = 40; *p* < 0.02). Importantly, 24 h after Bz injection, tumor MET-uptake was reduced significantly by 30–79% (mean reduction in TBR to 55 ± 18%; median of mean TBR 2.55, range 0.8–4.5, *p* < 0.02; *n* = 17) compared to the uptake at baseline in the same mouse. In the control group TBRs increased to a mean of 125 ± 34% (median of mean TBR 4.06, range 2.0–8.3, *p* = 0.432; *n* = 15) compared to the uptake at baseline in the same mouse.

**Figure 5:**
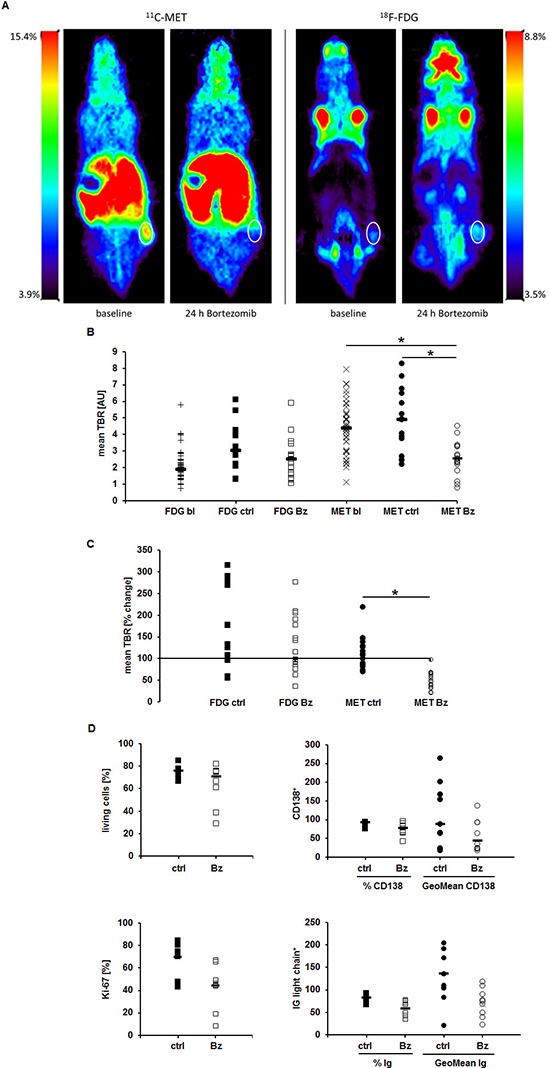
MET-PET reveals very early treatment response *in vivo* MM.1S tumors in Nod.Scid mice were imaged at baseline and 24 h after administration of Bz using MET- and FDG-PET, respectively. **(A)** Coronal views of an exemplary mouse. **(B)** Mean TBRs of FDG and MET at baseline (bl), in the control (ctrl) and treatment (Bz) group. **(C)** Relative change in tracer-uptake by tumors (mean TBR) in individual mice 24 h post treatment initiation compared to baseline. **(D)** FACS analyses evaluating dead cells (top left), CD138 (top right) and intracellular Ig light chain (bottom right) expression. Proliferation (bottom left) was assessed histologically by Ki-67 staining on paraffin embedded sections. Horizontal lines indicate the median in each group. Asterisk indicate statistical significance (*p* < 0.02). Differences in **(D)** are not statistically significant.

Using FDG-PET, no differences between TBRs of control (mean increase in TBR to 144 ± 79%; median of mean TBR 2.22, range 1.3–6.1, *n* = 17) or treatment group (mean increase in TBR to 141 ± 66%; median absolute TBR 2.53, range 1.0–5.9, *p* = 0.897; *n* = 15) could be detected (Figure 5A, 5B, 5C). Interestingly, molecular analyses of tumors resected after the last PET-scan did not show clear differences but only a slight tendency in the treatment-group to less living cells, proliferative activity, intracellular immunoglobulin light chain levels or CD138 expression (Figure 5D).

### Early reduction in MET uptake correlates with improved survival

**Figure 6:**
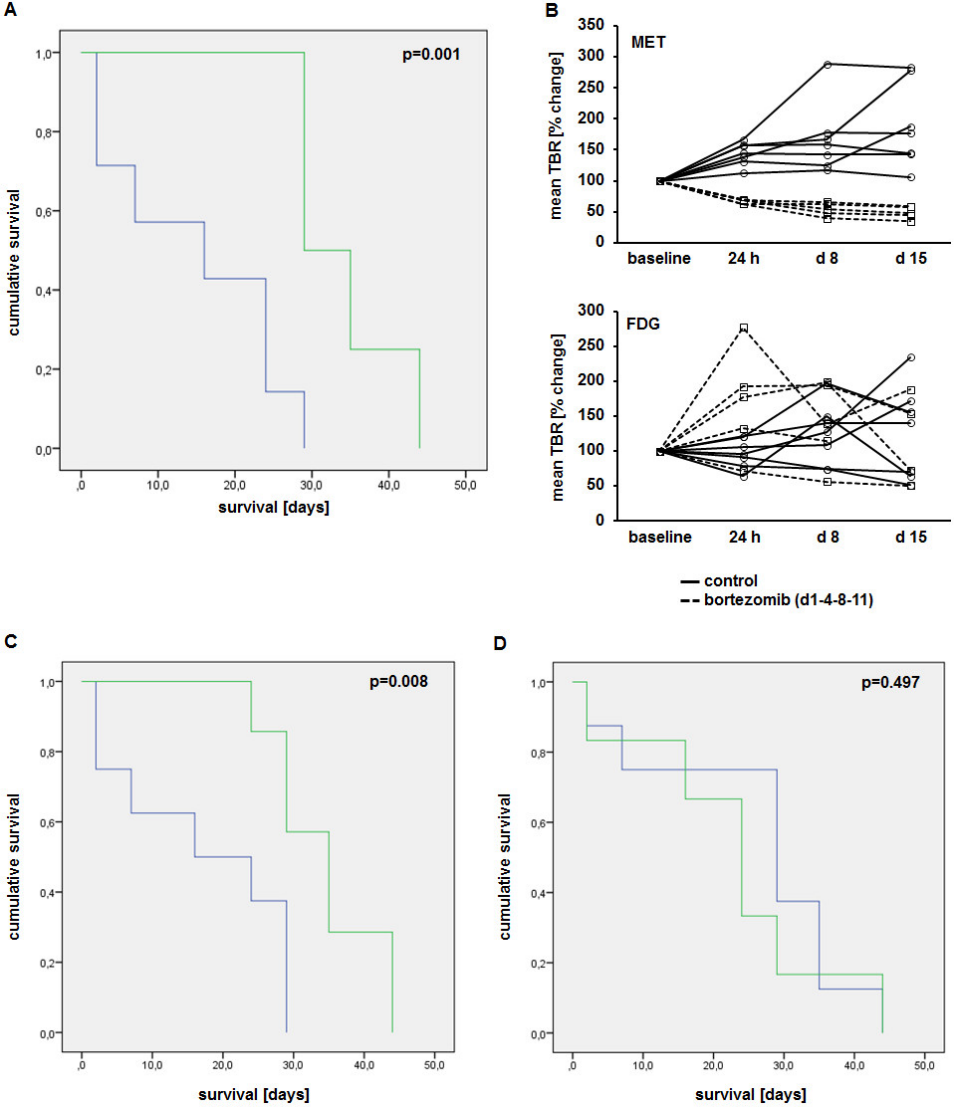
Early reduction in MET uptake correlates with improved survival **(A)** Kaplan-Meier-curve of animals treated with bortezomib (d1-4-8-11; *n* = 7; green) and control group (*n* = 8; blue). **(B)** Tumor-to-background ratios of MET (top) and FDG (bottom) uptake in treatment (dashed lines)- and control (solid lines) group at baseline, 24 h, 8d and 15d after treatment initiation. Correlation of changes in MET **(C)** and FDG **(D)** uptake 24 h post treatment initiation with survival (green, responders; blue, non-responders).

In a second line of experiments, xenografted mice received MET as well as FDG scans at baseline, 24 h, at d8 (before third Bz administration) and at d15 (4 days after completion of one Bz therapy cycle) post-treatment initiation. All mice were followed up until death or a human end point was reached. Treatment with Bz significantly reduced tumor burden (Supplementary Figure 3A, 3B) and improved survival of mice (median 34 days, range 30–39 days; compared to a median of 15 days, range 7–30 days, in the control-group; *p* = 0.001) (Figure 6A).

In all treated mice, MET uptake was markedly reduced 24 h post-treatment initiation (see Figure 5). MET-tumor-uptake remained constant or decreased only slightly in consecutive scans on d8 and d15. In the control group TBRs progressively increased over time (Figure 6B, top). In contrast, FDG scans revealed no discernible pattern and did not allow the separation in treatment-group and control (Figure 6B, bottom).

To investigate the impact of early reduction of MET-retention, changes in tracer uptake were correlated to survival and tumor burden. Mice with reduced MET uptake 24 h after treatment-initiation (cut-off: < 100% of TBR at baseline) had a significantly improved median survival of 35 days (range 29–40 d), compared to a median survival of 17 days (range 9–26d) in those animals with unchanged or higher tracer-retention (*p* = 0.008) (Figure 6C). In contrast, FDG-PET did not correlate with survival: while mice with reduced FDG-retention (24 h) had a median survival of 23 days (range 12–34d), mice with an increased uptake survived for a median of 26 days (range 16–36d; *p* = 0.497) (Figure 6D). Likewise, a reduced MET uptake, but not FDG uptake, 24 h after treatment-initiation correlated with a reduction in tumor burden after completion of one cycle bortezomib (d15; compared to baseline) (Supplementary Figure 3C). All mice with a reduced MET uptake at 24 h, a lower tumor burden at d15 and longer survival had been treated with bortezomib.

## DISCUSSION

This study aimed at the *in vivo* validation of the radiolabeled amino acid MET as radiotracer and biomarker for MM focussing on early assessment of treatment response and outcome prediction in comparison to FDG.

In MM cell lines and primary cells, initial MET uptake was higher than that of FDG. Although proteasome inhibition resulted in a reduction of both MET and FDG retention, MET uptake of treated cells was higher than that of FDG as well. This is well in line with our previously published data reporting high MET retention in MM cells (14). It might, at least in part, be explained by the observation that addition of the protein synthesis inhibitor cycloheximide reduced retention of MET in MM cells but not of FDG suggesting that MET is not only taken up but also incorporated into newly synthesized proteins (Supplementary Figure 4). By displaying relatively higher uptake in MM cells prior to and following treatment, MET might prove more sensitive in terms of detection of MM lesions *per se* and of changes in disease activity: visualization of MM manifestations with a low metabolism (usually missed by FDG) as well as of more subtle metabolic changes within a given lesion might be feasible using MET.

The proteasome inhibition-induced reduction in radiotracer uptake was correlated with alterations of other features of MM biology. We have already reported on a potential association of intracellular Ig light chains with initial MET uptake (14). In this series, extent of changes in radiotracer uptake and of reductions in cell proliferation or intracellular Ig light chains, respectively, could not be matched. However, CD138 expression on the cell surface showed a treatment-induced reversal of CD138^high^ to CD138^low^ MM cells which correlated with tracer uptake: the larger the reduction in tracer uptake, the lesser CD138 at the cell surface. The implication of this observation is not fully understood. CD138 is known to be an important factor for bone marrow homing of MM cells ((15) and references therein). A decrease of CD138 levels might thus be a direct result of proteasome inhibition, thereby promoting tumor cell migration from the BM niche into the periphery where immunosurveillance is more effective (16, 17).

Taken together, our *in vitro* data suggest superiority of MET to FDG regarding MM detection, monitoring of treatment response and a possible link to underlying tumor biology. Although FDG has been reported to be of prognostic value in therapy assessment (10, 11, 18, 19), given our data, MET seems to be a more versatile marker.

This notion is further corroborated by *in vivo* experiments in xenotransplanted Nod.Scid mice. Well in line with our findings in MM cell lines and primary cells ((14) and Figure 1), μPET-imaging with MET prior to treatment resulted in much higher tumor-to-background ratios underlining its superior sensitivity in comparison to FDG. Most importantly, treatment and control groups could clearly be differentiated with MET but not with FDG. Only MET was capable to detect very early tumor response as early as 24 h after initiation of proteasome inhibitor treatment; neither FDG-uptake nor any other tumor marker (Ki-67/proliferation, CD138, Ig light chains) was affected at that time. As MET intensity robustly dropped by ≥ 30% the early reduction of TBRs observed here is likely to reflect a true response (20–22). In addition, a metabolic response in 24 h PET-imaging compared to baseline significantly correlated with prolonged survival and reduced tumor burden. Thus, MET not only seems to improve MM diagnosis and therapy monitoring but also to be a radiotracer providing predictive potential regarding response (discrimination of responders and non-responders) and outcome. The former notion is also supported by other studies demonstrating a clinical value of MET-PET with an equal or greater number of MM lesions detected by MET *vs*. FDG (23–25). Interestingly, a very recent study demonstrated a correlation of large amino acid transporter 1 (LAT1) expression on patient-derived MM cells with increased proliferative activity of these cells and a worse prognosis (26). LAT1 is the major transmembrane-transporter for MET and an association between LAT1 expression and ^11^C-MET uptake has been shown in gliomas (27). Thus, it is appealing to speculate about a potential connection of MET-uptake and patient prognosis. In translation to the human disease, very early response assessment might impact on patient management by termination of ineffective treatment regimens, thereby optimizing anti-myeloma drug activity and minimizing futile adverse effects. Additionally, early MET-PET might indicate subjects with an overall very good response but a single or few remaining lesions with persisting high metabolism after one week of treatment. These patients might be candidates for additional local treatment such as radiation therapy, enabling local disease control. Thus, very early PET imaging using radiolabeled amino acids such as MET would establish a novel approach for treatment individualization, allowing for therapy initiation and adjustments earlier than any other existing method, even outperforming analysis of free light chains (28). A pilot trial investigating the performance of very early treatment response MET-PET in patients with MM is highly warranted to further investigate the usefulness of PET imaging in this regard. Potential drawbacks of the amino acid tracer concerning reduced sensitivity within the liver and pancreas due to high physiologic uptake can be neglected, given the very rare involvement of these organs in this disease and the broad availability of combined PET/CT imaging. Access to an on-site cyclotron could represent a limiting factor for the widespread use of ^11^C-MET PET in daily clinical practice.

Concluding, our results suggest that MET is superior to FDG in detecting MM manifestations, in assessing response to anti-MM treatment and might provide a link to underlying tumor biology. Monitoring of response at a very early time seems feasible exclusively with MET-PET. Moreover, early changes in MET retention have predictive potential regarding response and survival. Thus, MET uptake might serve as a biomarker for MM, opening the possibility for individualized therapies and promptly adapted treatment strategies.

## MATERIALS AND METHODS

### Ethics statement

All experiments involving human material were approved by the ethics committee of the University of Wuerzburg (#192/12). Bone marrow biopsies from patients diagnosed with MM were taken after obtaining informed written consent from each patient.

Animal studies were performed in agreement with the Guide for Care and Use of Laboratory Animals published by the US National Institutes of Health (NIH Publication No. 85–23, revised 1996) and in compliance with the German law on the protection of animals (license 55.2–2531.01–65/12).

### Cell culture and treatment

The human myeloma cell lines INA-6 (provided by Dept. of Hematology, University Hospital Wuerzburg), OPM-2 (DSMZ no. ACC50) and MM.1S (ATCC no CRL-2974) were cultured and tested for contamination with mycoplasma as described previously (14). Cell line identity was confirmed at the DSMZ (July 2013). Where indicated, cells were treated for 48 h with bortezomib (Velcade^®^), ixazomib (MLN9708) or carfilzomib (Kyprolis^®^). All drugs were obtained from Selleckchem (Munich, Germany). Concentrations used were established by adding various amounts of the respective compound to the cells and assessing the extent of cell death induced. For treatment, the respective concentration resulting in 50% dead cells was chosen (Table 1):

**Table 1:** Concentration of drugs used for the treatment of myeloma cell lines

	INA-6	MM.1S	OPM-2
**bortezomib**	2.5 nM	2.0 nM	2.5 nM
**ixazomib**	28.5 nM	36 nM	20 nM
**carfilzomib**	6.0 nM	6.0 nM	7.0 nM

For inhibition of protein synthesis, cyclohexamide (Sigma-Aldrich, Taufkirchen, Germany) was used at a concentration of 50 μg/mL for 2 h.

### Isolation of CD138^+^-plasma cells

CD138^+^-plasma cells were isolated from bone marrow aspirates of patients diagnosed with MM as previously described (14). Uptake experiments were performed as described below but with 100 000 cells/sample and incubation with 1*10^6^ counts per minute (cpm) of either radiotracer for 60 min. Where indicated, 2.0 nM bortezomib was added to the sample 24 h prior to the uptake study.

### Flow cytometric analyses

Single cell suspensions were stained with fluorochrome conjugated antibodies against hCD138^+^-APC (Syndecan; clone B-B4), anti-hIg kappa light chain-APC (clone IS11–24D5) or anti-hIg lambda light chain-FITC (clone IS7–24C7; all antibodies from Miltenyi, Bergisch-Gladbach, Germany) and analyzed with a BD FACSCalibur flow cytometer using the BD CellQuest software (Beckton Dickinson, Heidelberg, Germany) according to the manufacturer's instructions.

### Cell proliferation assay (cell culture)

Proliferation was assessed by labeling cells with 1 μM carboxyfluorescein diacetate succinimidyl ester (CFSE; Invitrogen, Karlsruhe, Germany) in 3% FCS/PBS for three minutes at room temperature. Cells were seeded at a density of 1*10^5^ cells/well on a 96-well plate, allowed to grow for 48 h and were then harvested and resuspended in 300 μl propidium iodide (PI) solution (2.5 μg PI (Sigma-Aldrich, Taufkirchen, Germany)/3% FCS/PBS). Proliferation of living cells was measured by FACS in the FL-1 channel as the decrease in CFSE fluorescence intensity.

### Ki-67 staining of paraffin embedded tumor tissue

Tumors were resected and immediately embedded in Tissue-Tek^®^ OCT Compound (PLANO, Wetzlar, Germany) for cryostat sectioning (Microm HM-500 M Cryostat; Thermo-Scientific, Dreieich, Germany). 6 μm-sections were fixed with absolute acetone, blocked with 0.2% BSA (Sigma-Aldrich, Taufkirchen, Germany)/2% donkey serum (abcam, Cambridge, UK)/PBS and stained with rabbit-anti-Ki-67 antibody (dilution 1:100; clone SP6; abcam, Cambridge, UK) for 1 h. An Alexa Fluor^®^ 555 donkey-anti-rabbit IgG antibody (dilution 1:800, 1 h; Molecular probes, Darmstadt, Germany) was used for detection. Cell nuclei were stained with dapi (dilution 1:1000, 10 min; Molecular probes, Darmstadt, Germany). Slides were coverslipped with VECTASHIELD^®^ Mounting Medium (Biozol, Eching, Germany). For analysis, all cells in a field of view at 40x magnification were counted using Image J Software (http://imagej.nih.gov/ij/) and the percentage of proliferating (Ki-67 positive) cells was determined. In total, five fields of view were evaluated and mean and standard deviation were calculated.

### CD138 (Syndecan) ELISA

CD138 released into medium was quantified by sandwich-ELISA using the Syndecan (CD138) Human ELISA Kit (abcam, Cambridge, UK) according to the manufacturer's instructions. Samples were measured undiluted and in duplicates.

### Synthesis of ^18^F-FDG (FDG) and ^11^C-MET (MET)

Radiopharmaceuticals were produced in-house with a 16 MeV Cyclotron (GE PETtrace 6; GE Healthcare, Milwaukee, WI, USA) as described previously (14).

### Cellular uptake experiments

Sub-confluent cell cultures were harvested and adjusted to a concentration of 400 000 cells/500 μL PBS per sample. Radioactive substances were diluted to 1*10^6^ cpm/50 μL PBS. After addition of 1*10^6^ cpm, samples were incubated for various time intervals up to 120 min at 37°C. Tracer uptake was stopped by incubation on ice, followed by washing twice with PBS to remove residual radioactivity. Intracellular radioactivity was quantified using a semi-automated gamma-counter (Wallac 1480-Wizard, Perkin Elmer, Rodgau, Germany). All samples were measured in triplicates. Background activity- and decay-corrected data were expressed as cpm per 1000 cells.

### Animal studies

NOD.CB17-*Prkdc^scid^*/NCrHsd mice were bred at the animal facility at the Center of Experimental and Molecular Medicine (ZEMM) of the University of Wuerzburg. Animals received water and food *ad libitum*; mice were housed in filter-top cages at ambient temperature with a light/dark cycle of 12/12 h. Pathogen status was assessed according to FELASA B protocol on a regular basis. 5*10^6^ MM.1S cells in 100 μl PBS were injected subcutaneously into the flank of approximately 8 week old animals. Mice were visually inspected daily and tumor growth was monitored using a shifting caliper. Imaging experiments were initiated when tumor size reached 100–200 mm^3^. Mice (*n* = 40) were randomized into control (0.9% NaCl) and treatment groups (*n* = 20 each; group sizes corresponded to the biometrical calculations provided in the relevant animal license). The latter received 1 μg bortezomib/g body weight in 100 μl 0.9% NaCl intraperitoneally at day 1, 4, 8 and 11. All mice received a PET-examination with MET, followed (4–5 h later) by a scan with FDG at baseline (day 0) and 24 h after treatment-initiation. Where indicated, MET and FDG scans were repeated at day 8 and day 15. Radiotracers (7–9 MBq/mouse) were injected into the tail vein of anesthetized (2% isoflurane; Abbott, Wiesbaden, Germany) animals using a veterinary anesthesia system (Vetland Medical, Louisville, KY, USA). For MET-PET, scanning was initiated 5 min post tracer-injection and lasted for 10 min; FDG images were acquired 60 min post-injection for 15 min. Owed to the short half-life of ^11^C-MET (20 min), acquisition time for MET-PET was shortened to 10 min to allow analysis of all mice in a given experimental cohort with the same batch of MET. Tumor lesions were imaged using a small-animal PET scanner (Inveon; Siemens Medical Solutions, Erlangen, Germany) and images were reconstructed using ordered subset expectation maximization 2D (OSEM 2D) algorithm. Mean tumor-to-background ratios (TBR) were determined by drawing a volume of interest (VOI; 3 × 3 × 3 pixels) around individual tumor lesions and healthy soft tissue in the contralateral flank (background) using ‘A Medical Image Data Analysis Tool’ (AMIDE)-software (http://amide.sourceforge.net/). Background contrast for MET and FDG was similar (MET: median 49346 AU; FDG: median 42703 AU).

### Statistical analysis

Statistical analyses were performed using PASW Statistics software (version 22.0; SPSS, Inc. Chicago, IL) or Microsoft Excel. Quantitative values were expressed as mean ± standard deviation or median and range as appropriate. Comparisons of related metric measurements were performed using Mann-Whitney-*U* test. Survival probabilities were calculated according to the Kaplan-Meier method and the log-rank test was used for statistical comparison of survival curves between independent subgroups. All statistical tests were performed two-sided and a *p*-value < 0.05 was considered to indicate statistical significance.

## SUPPLEMENTARY FIGURES AND TABLE


