# Supplementary material for: 11C-Methionine-PET: A novel and sensitive tool for monitoring of early response to treatment in multiple myeloma
Source: Oncotarget. 2015 Jan 20;6(10):8418–29. doi: 10.18632/oncotarget.3053 (PMC4480763; doi:10.18632/oncotarget.3053)
Supplement: Supplementary file 1 [file oncotarget-06-8418-s001.pdf]

SUPPLEMENTARY FIGURES AND TABLE

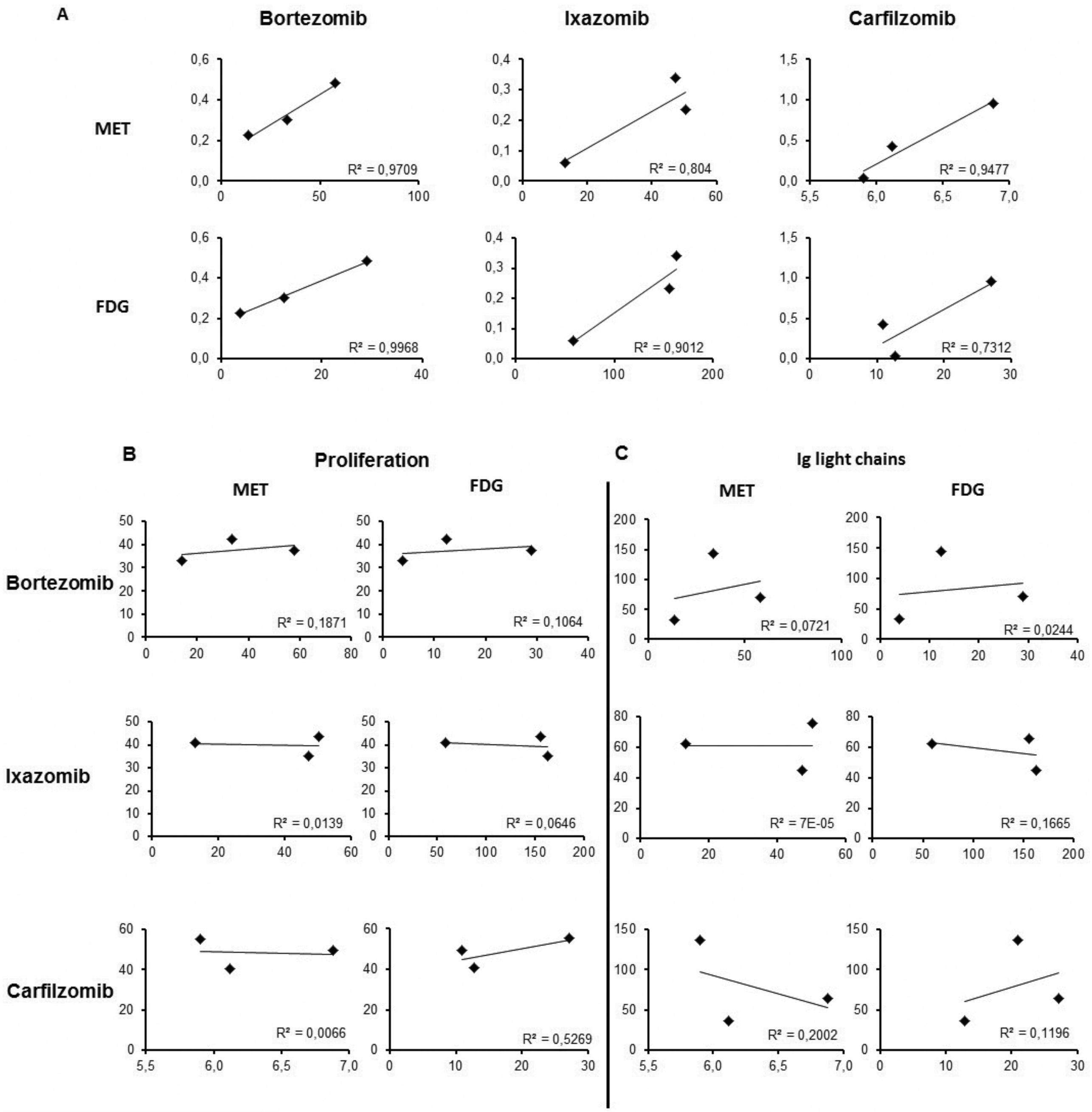

Supplementary Figure 1: Correlation between the extent of treatment-induced alterations in CD138 expression, proliferation and Ig light chain levels, respectively, with changes in tracer-uptake in MM cell lines. Mean CD138^{high}/CD138^{low} ratio (%) **(A)**, reduction in proliferation (CFSE; % reduction GeoMean) **(B)** and intracellular Ig light chain levels (% reduction GeoMean) **(C)** *vs*. change (%) in MET-uptake and FDG-uptake after 60 min.

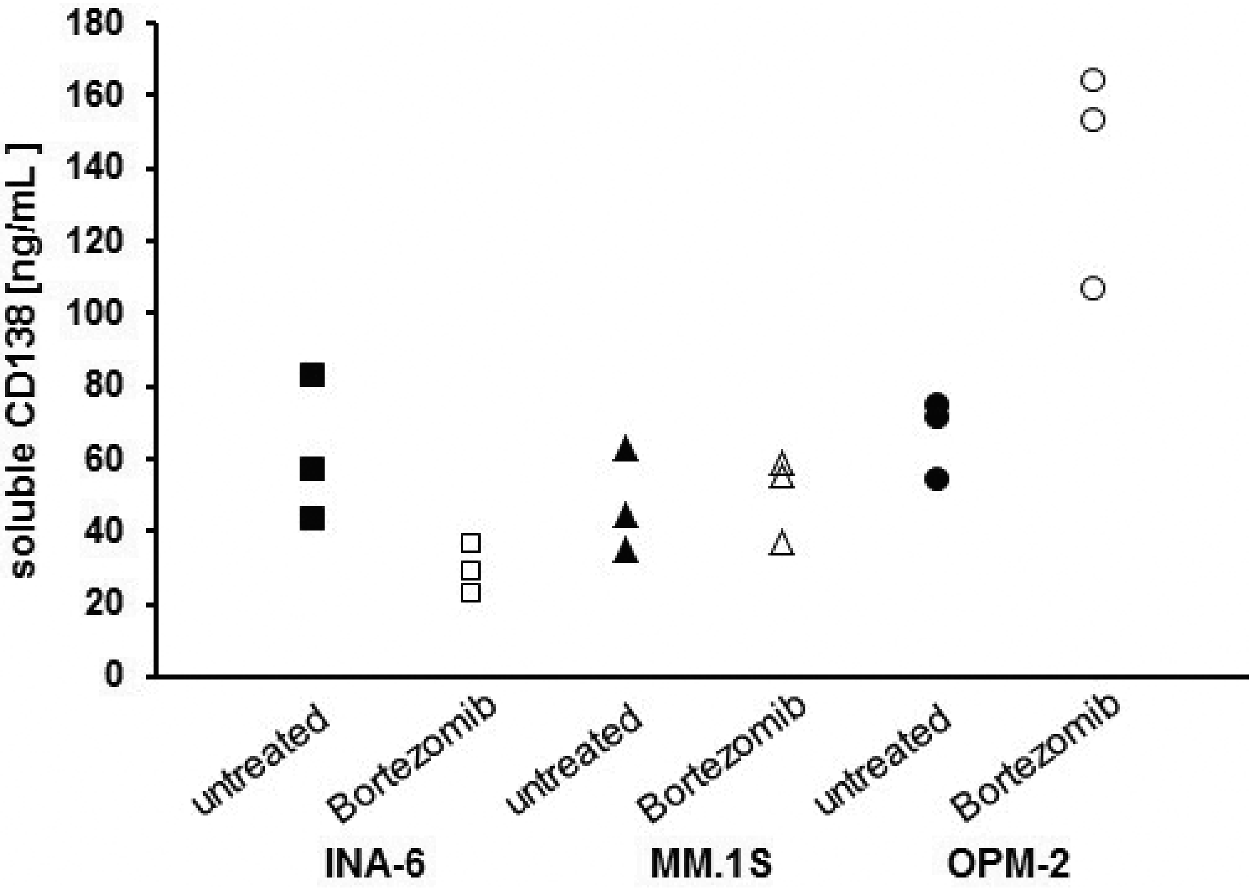

Supplementary Figure 2: Loss of CD138 from the cell surfaces can only in parts be explained by enhanced CD138-shedding. Quantification of soluble CD138 in the medium of MM cell lines cultured for 48 h in the presence or absence of 2 nM Bz using Syndecan-ELISA. Three individual experiments are shown.

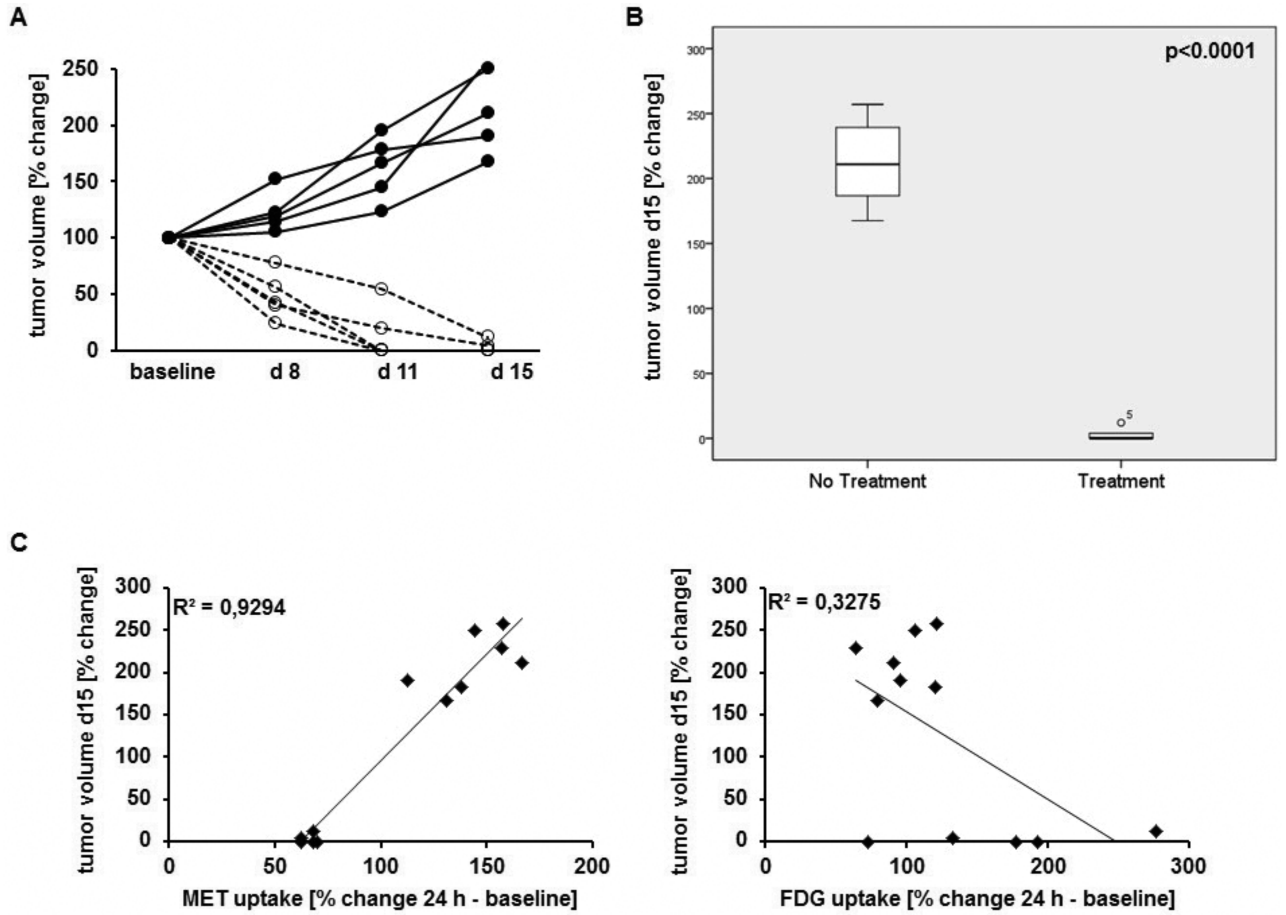

Supplementary Figure 3: Tumor burden over time. (A) Change in tumor volume over time (baseline – d8 – d11 – d15) in mice treated with bortezomib (dashed lines) and control animals (solid line). Tumor size was measured at the indicated time points with a shifting caliper and calculated as (length * width^2)/2. Data are expressed as change (%) compared to tumor volume at baseline PET examination. **(B)** Tumor volume at day 15 (3 days after end of first cycle bortezomib) in control (left) and treatment (right) group given as percent of tumor volume before therapy. Shown are median and range. **(C)** Correlation of change in MET (left) and FDG (right) uptake 24 h post treatment initiation with change in tumor volume at day 15, both compared to baseline.

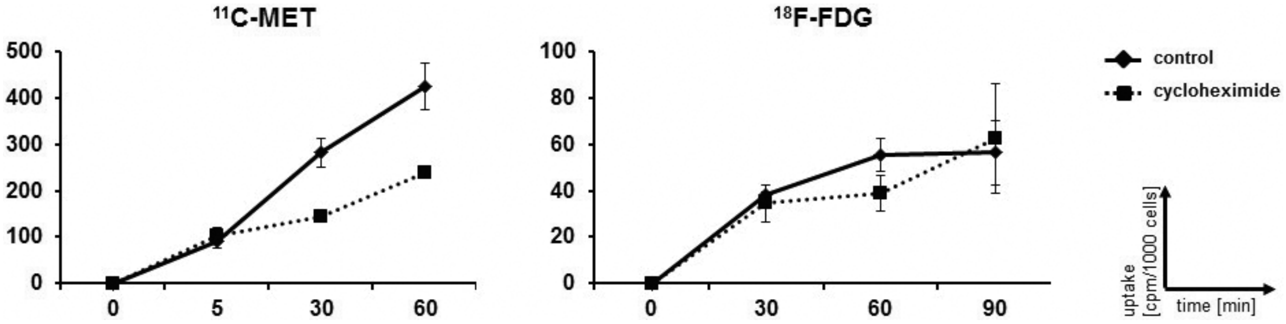

Supplementary Figure 4: Differences in MET and FDG uptake following treatment with proteasome-inhibitors might be explained by decreased incorporation of MET into proteins. MM.1S cells were or were not treated with cycloheximide for 2 h before intracellular radioactivity following incubation with MET (top) or FDG (bottom) was quantified using a gamma-counter. Relative uptake of background- and decay-corrected triplicate-samples was expressed as cpm per 1000 cells (mean ± sem; $n = 3$).

Supplementary Table 1: Patient' characteristics

patient no.	age	sex	diagnosis	Ig	DS* stage	initial diagnosis
**1**	75	m	MM	IgG κ	IIIB	01/2014
**2**	69	m	MM	IgAλ	IIIA	06/2012
**3**	56	m	MM	IgG κ	IIIA	06/2011
**4**	65	f	plasmacytoma	n/a	n/a	01/2014
**5**	65	m	MM	IgG κ	IIIA	11/2013
**6**	67	f	MM	IgA κ	IIIA	03/2014
**7**	61	f	MM	IgG κ	IIIA	03/2004
**8**	79	m	MM	κ light chains	IIIB	08/2006

*DS = Durie/Salmon stage
